# Oral Unsaturated Fat Load Impairs Postprandial Systemic Inflammation in Primary Hypercholesterolemia Patients

**DOI:** 10.3389/fphar.2021.656244

**Published:** 2021-04-20

**Authors:** Aida Collado, Elena Domingo, Patrice Marques, Eva Perello, Sergio Martínez-Hervás, Laura Piqueras, Juan F. Ascaso, José T. Real, Maria-Jesus Sanz

**Affiliations:** ^1^ Department of Pharmacology, Faculty of Medicine and Odontology, University of Valencia, Valencia, Spain; ^2^ Institute of Health Research INCLIVA, University Clinic Hospital of Valencia, Valencia, Spain; ^3^ CIBERDEM-Spanish Biomedical Research Center in Diabetes and Associated Metabolic Disorders, Carlos III Health Institute, Madrid, Spain; ^4^ Department of Medicine, Faculty of Medicine and Odontology, University of Valencia, Valencia, Spain

**Keywords:** primary hypercholesterolemia, oral unsaturated fat load, systemic inflammation, platelet activation, leukocytes, inflammatory mediators, endothelial dysfunction

## Abstract

**Context: **Primary hypercholesterolemia (PH) is a lipid disorder characterized by elevated levels of cholesterol and low-density lipoprotein (LDL). Low-grade systemic inflammation is associated with PH, which might explain the higher incidence of cardiovascular diseases in this setting.

**Objective: **To evaluate the effect of an oral unsaturated fat load (OUFL) on different immune parameters and functional consequences in patients with PH in postprandial state.

**Design: **A commercial liquid preparation of long-chain triglycerides (Supracal^®^; *ω*6/*ω*3 ratio >20/1, OUFL) was administered to 20 patients and 10 age-matched controls. Whole blood was collected before (fasting state) and 4 h after administration (postprandial state). Flow cytometry was employed to determine platelet and leukocyte activation, and the levels of circulating platelet-leukocyte aggregates. Soluble markers were determined by ELISA, and the parallel-plate flow chamber was employed to study leukocyte adhesion to the dysfunctional arterial endothelium.

**Results:** The PH group had a lower percentage of activated platelets and circulating type 1 monocytes, and blunted neutrophil activation after the OUFL, accompanied by a significant increase in the percentage of regulatory T lymphocytes. In this group, the OUFL led to a significant impairment of leukocyte adhesion to the dysfunctional [tumor necrosis factor α (TNFα)-stimulated] endothelium and reduced the plasma levels of soluble P-selectin, platelet factor-4 (PF-4)/CXCL4, CXCL8, CCL2, CCL5, and TNFα.

**Conclusion: **The OUFL has a beneficial impact on the pro-thrombotic and pro-inflammatory state of PH patients and might be a promising macronutrient approach to dampen the systemic inflammation associated with PH and the development of further cardiovascular events.

## Introduction

Cardiovascular disease (CVD) remains one of the leading causes of death in Western societies (Benjamin et al., 2017; Wilkins et al., 2017). The main risk factors for CVD include age, hypertension, obesity, diabetes *mellitus* and high circulating levels of low-density lipoprotein (LDL) (Wong et al., 2016). Despite improvements in primary prevention and the identification of new pharmacological agents to reduce blood cholesterol levels, the adoption of unhealthy lifestyle habits has led to an increased incidence of hypercholesterolemia, diabetes *mellitus* and obesity, which in turn has increased the incidence and prevalence of atherosclerosis–the predominant cause of CVD (Hedrick 2015; Zimmer et al., 2015).

Primary hypercholesterolemia (PH) is a metabolic disorder characterized by elevated plasma levels of cholesterol, in particular, LDL and apolipoprotein B (apoB). PH is genetically heterogeneous and includes both familial hypercholesterolemia (FH, prevalence of 1:200–1:500) and non-familial polygenic hypercholesterolemia, which is far more frequent (Langslet et al., 2015). The marked increase of LDL in the bloodstream in PH can trigger the development of atherosclerotic plaques in arteries, increasing the risk of premature coronary artery disease (Defesche et al., 2017), which for untreated hypercholesterolemia can be 20% higher than in control subjects (Sniderman et al., 2014). Yet, it has become more evident that systemic inflammation seems to be the main driver of premature atherosclerosis and its complications, together with elevated LDL plasma levels (Catapano et al., 2017). Indeed, several studies have demonstrated that low-grade systemic inflammation is associated with PH, which might explain the higher incidence of CVD in these patients beyond elevated LDL cholesterol plasma values (Langslet et al., 2015; Barale et al., 2018; Collado et al., 2018a).

The oral unsaturated fat load (OUFL) test is a standardized method to study postprandial lipemia that allows the evaluation of the relationship between fatty acids and different parameters in a non-fasting situation (Garcia-Garcia et al., 2019). In this regard, an OUFL challenge in patients with FH was found to modulate oxidative/antioxidative status, reducing overall oxidative stress (Pedro et al., 2013; Cortes et al., 2014) and the levels of some atherosclerosis-related chemokines (Cortes et al., 2016). The postprandial effect of unsaturated fats on the immune state in patients with PH has, by comparison, received much less attention.

We previously reported the existence of low-grade systemic inflammation in patients with PH, which is accompanied by a pro-thrombotic state driven by exacerbated platelet activation and endothelial dysfunction, likely explaining the higher incidence of further CVD (Collado et al., 2018a). In the present study, we tested whether a 4 h OUFL challenge in patients with PH favorably impacts different immune and functional outcomes. A secondary aim was to examine whether the inflammatory state and tumor necrosis factor α (TNFα)-induced endothelial leukocyte adhesion, a key feature of endothelial dysfunction, was improved after this time. To do this, we enrolled age-matched controls and untreated, primarily diagnosed patients with PH.

## Materials and Methods

The present study was performed following The Code of Ethics of the World Medical Association outlined in the Declaration of Helsinki of 1975 and revised in 1983 for experiments that involve human subjects. The Clinical Research Ethics Committee of the University Clinic Hospital of Valencia, Spain approved the protocol for this study. All subjects signed the appropriate written informed consent to participate in the study, and the privacy rights of subjects were always observed.

This manuscript is in line with the Recommendations for the Conduct, Reporting, Editing, and Publication of Scholarly Work in Medical Journals and aims for the inclusion of representative human populations (gender, age, and ethnicity) as per those recommendations.

### Cell Culture

Human umbilical artery endothelial cells (HUAEC) were isolated from human umbilical cords by collagenase treatment as previously described (Jaffe et al., 1973). Cells were maintained in human endothelial cell basal medium-2 supplemented with endothelial growth medium-2 (both from Lonza Group, Basel, Switzerland) with 10% fetal bovine serum (Biowest, Nuaillé, France). Cells were grown to confluence up to passage 1 to preserve endothelial features. Before every experiment, cells were incubated for 24 h in medium containing 2% fetal bovine serum.

### Human Study Populations

Twenty patients with PH and 10 age-matched controls were included in the study, and all were recruited by the Endocrinology and Nutrition Service at the University Clinic Hospital of Valencia, Spain.

Subjects fulfilled the diagnosis, inclusion and exclusion criteria to be considered for the study (Collado et al., 2018a), as described in Table 1.

**TABLE 1:** Diagnosis, inclusion and exclusion criteria for patients and age-matched controls to participate in the study (Collado et al., 2018a)^1^.

Diagnosis criteria (PH patients)	Inclusion criteria (age-matched controls)	Exclusion criteria (both groups)
TC > 260 mg/dl and/or LDL >160 mg/dl	TC < 200 mg/dl and apoB <120 mg/dl	CVD, hypertension, diabetes, chronic diseases or cancer
TG < 150 mg/dl	TG < 150 mg/dl	Smoking Alcohol consumption >30 g/day
	Fasting plasma glucose <100 mg/dl	Renal or hepatic insufficiency and hypothyroidism
	The absence of dyslipidemia, CVD or diabetes	Pregnancy or lactation
		Infection, inflammatory disease (including asthma, allergy, and autoimmune deficiency) or drugs able to alter inflammation six weeks before the study

^1^ apoB, apolipoprotein B; CVD, cardiovascular disease; LDL, low-density lipoprotein; PH, primary hypercholesterolemia; TC, total cholesterol; TG, triglycerides.

### Oral Unsaturated Fat Load Administration

The study started at 8:30 a.m. and blood was drawn by venipuncture after a fasting period of at least 12 h. Anthropometric parameters and blood pressure were measured using standardized procedures: body mass index (BMI; kg/m^2^), waist circumference (midpoint between the edge lower rib and iliac crest; cm) and blood pressure (mmHg). Blood samples from PH patients and age-matched controls were taken and collected in Vacutainer^®^ blood collection tubes containing sodium citrate (3.2%), or in Vacutainer^®^ PST^™^ II tubes with lithium/heparin (17 IU/ml) as anticoagulant agents (BD Biosciences, San Jose, CA, United States) and subsequently subjected to different analytical determinations including complete biochemistry with glycemic and lipid profile, and quantification of creatinine as a measure of renal function.

After the first blood sampling (time 0; T0), both patients and controls ingested a commercial OUFL of 25 g of a high-fat meal per m^2^ of body surface area, prepared with a commercial long-chain triglycerides (TG) liquid preparation (Supracal^®^; SHS International, Liverpool, United Kingdom). Each 100 ml of the formula contained 50 g of fat (450 kcal), of which 9.6 g was saturated, 28.2 g monounsaturated and 10 g polyunsaturated, with a *ω*6/*ω*3 ratio >20/1. Fatty acid content and the complete composition are detailed in Supplementary Table S1, as explained previously (Pedro et al., 2013). Subjects were only allowed to drink mineral water during the test. Blood samples were taken before and 4 h after the OUFL challenge for the various measurements.

### Flow Cytometry

Whole blood was stained with saturated amounts of antibodies as indicated by the manufacturers. A Fc blocker was not used since flow cytometry studies have been performed in whole blood. Under these circumstances it is widely accepted that whole blood contains enough amounts of IgGs to block Fc receptor. All samples were analyzed in a FACSVerse™ flow cytometer (BD Biosciences) and data analyzed with FlowJo® v10.0.7 software (FlowJo LLC, Ashland, OR).

#### Measurement of Platelet Activation

To assess platelet activation, PAC-1^+^ platelets and P-selectin expression were assessed by flow cytometry. Samples of citrated blood were diluted in glucose buffer [1:10; 1 mg/ml glucose in phosphate-buffered saline (PBS) containing 0.35% bovine serum albumin (BSA); Sigma-Aldrich, St. Louis, MO, United States] (Murugappa and Kunapuli, 2006), and then incubated for 30 min with a 5-carboxyfluorescein (CF)-Blue™-conjugated monoclonal antibody (mAb) against human CD41 (clone HIP8, IgG_1_; Immunostep, Salamanca, Spain), a fluorescein isothiocyanate (FITC)-conjugated mouse mAb against human PAC-1 (clone PAC-1, IgM; BD Biosciences) or with an allophycocyanin (APC)-conjugated mAb against human P-selectin (CD62P, clone HI62P, IgG_1_; Immunostep).

The identification of the platelet population was achieved based on their morphology according to size and complexity [side scatter (SSC) *vs.* forward scatter (FSC)]. They were subsequently selected as the CD41^+^ population and expressed as the percentage of positive platelets, as illustrated in the gating strategy in Supplementary Figure S1.

#### Leukocyte Subpopulation Studies

##### Neutrophils

To assess the polymorphonuclear population present in blood samples, a high SSC and staining with a Pacific Blue (PB)™-conjugated mouse mAb against human CD45 (clone HI30, IgG_1_; BioLegend, San Diego, CA, United States) were combined to select the neutrophil population. Analysis with a FITC-conjugated mouse mAb against human CD16 (clone 3G8, IgG_1_; BD Biosciences) was first performed to distinguish them, and the possible presence of platelets was studied using two different experimental approaches. First, heparinized whole blood was analyzed to detect neutrophils attached to platelets. Second, and in parallel, blood samples were incubated with 10 mM ethylenediaminetetraacetic acid (EDTA; PanReac AppliChem GmbH, Darmstadt, Germany), for 15 min at 37°C to promote platelet dissociation, as described (Postea et al., 2012). In both cases, a phycoerythrin (PE)/Cy™7-conjugated mouse mAb against human CD41 (clone HIP8, IgG_1_; BioLegend) was used to determine the neutrophil-platelet aggregates (Supplementary Figure S2). The activation state in this cell population was determined with an APC-conjugated mouse mAb against human integrin CD11b (clone ICRF44, IgG_1_; BioLegend) and a PE-conjugated mAb against human CD69 (clone FN50, IgG_1_; Immunostep).

##### Monocytes

An APC-conjugated mouse mAb against human CD14 (clone 47-3D6, IgG_2A_; Immunostep) was used to distinguish monocytes from other leukocyte types and positive cells were selected. To identify the different types of monocytes, the CD14 population was confronted with a FITC-conjugated mouse mAb against human CD16 (clone 3G8, IgG_1_; BD Biosciences) and a brilliant violet BV421™-conjugated mouse mAb against human CD192 (CCR2, clone K036C2, IgG_2A_; BioLegend) (Supplementary Figure S3) (Collado et al., 2018a). Consequently, three subpopulations of monocytes were distinguished as described in Supplementary Table S2. Monocyte-platelet aggregates were determined following the same strategy as explained above.

A PE-conjugated mouse mAb against human integrin CD11b (clone CBRM1/5, IgG_1_; BioLegend) was used to determine the activation state of these cell populations. Similarly, fractalkine/CX_3_CL1 receptor (CX_3_CR1) expression was also determined in these three monocyte subpopulations using a PE-conjugated rat mAb against human CX_3_CR1 (clone 2A9-1, IgG_2B_; BioLegend).

##### T Lymphocytes

The markers CD3 (T lymphocytes), CD4 (T helper lymphocytes; Th), and CD8 (cytotoxic T lymphocytes) were used to identify T lymphocyte populations within the leukocytes present in peripheral blood. The gating strategy consisted in a selection through an APC-conjugated mouse mAb against human CD3 (clone 33-2A3, IgG_2A_; Immunostep), a V450-conjugated mouse mAb against human CD4 (clone RPA-T4, IgG_1_; BD Biosciences), and a FITC-conjugated mouse mAb against human CD8 (clone SK1, IgG_1_; BD Biosciences) (Supplementary Figure S4). After the identification of the different subpopulations, the possible contribution of platelets and T lymphocyte activation was studied as described above.

##### T Helper Lymphocytes

To select the different Th lymphocyte subpopulations, we first used a PerCP/Cy™5.5-conjugated mouse mAb against human CD4 (clone RPA-T4, IgG_1_; BD Biosciences). Once detected, Th subpopulations were identified (as shown in Supplementary Table S3) with an Alexa Fluor® 488-conjugated mouse mAb against human CD183 (CXCR3, clone 1C6/CXCR3, IgG_1_; BD Biosciences) and a BV421™-conjugated mouse mAb against human CD196 (CCR6, clone 11A9, IgG_1_; BD Biosciences) (Supplementary Figure S5).

Heparinized whole blood incubated or not with EDTA was employed to determine the Th lymphocyte-platelet complexes or platelet-free Th lymphocytes using a PE/Cy™7-conjugated mouse mAb against human CD41 (clone HIP8, IgG_1_; BioLegend). The activation state was determined using an APC-conjugated mouse mAb against human CD69 (clone FN50, IgG_1_; BD Biosciences).

##### Regulatory T Lymphocytes (Treg)

To identify the Treg population, we used a human regulatory T cell cocktail (BD Pharmingen™ Human Regulatory T Cell Cocktail; BD Biosciences). Th lymphocytes were first identified with the marker CD4 (FITC-conjugated mouse mAb, clone SK3, IgG_1_; BD Biosciences). Then, an Alexa Fluor® 647-conjugated mouse mAb against human CD127 (clone HIL-7R-M21, IgG_1_; BD Biosciences) and a PE/Cy™7-conjugated mouse mAb against human CD25 (clone 2A3, IgG_1_; BD Biosciences) were used to determine natural Treg cells (Supplementary Figure S6). As previously indicated, two experimental approaches were carried out in parallel: one with heparinized whole blood and the other with EDTA-treated blood, to determine the percentage of Treg lymphocyte-platelet aggregates. For this purpose, a CF-Blue™-conjugated mAb against human CD41 (clone HIP8, IgG_1_; Immunostep) was employed.

### Quantification of Soluble Metabolic and Inflammatory Markers

Human soluble adiponectin, leptin and ghrelin, as well as cytokines and chemokines including interleukin (IL)-6, IL-8/CXCL8, IL-10, IL-12, growth-regulated oncogene α (GROα)/CXCL1, fractalkine/CX_3_CL1, TNFα, interferon γ (IFNγ), monocyte chemoattractant protein-1 (MCP-1)/CCL2, regulated upon activation, normal T cell expressed and secreted (RANTES)/CCL5, platelet factor-4 (PF-4)/CXCL4 and soluble P-selectin (sP-selectin) were measured in plasma samples using commercial ELISA (Human DuoSet® ELISA; R&D Systems, Inc., Minneapolis, MN, United States). Results are expressed as pg/mL or ng/mL of mediator in plasma.

### Leukocyte-Endothelial Cell Interactions Under Flow Conditions

To measure leukocyte adhesion, HUAEC were seeded on 35-mm-diameter pre-treated culture plates. Once confluence was reached, plates were placed in a parallel flow chamber (GlycoTech, Gaithersburg, MD, United States). Whole blood from subjects was diluted 1:10 in Hanks balanced salt solution (Lonza Group) and perfused across HUAEC monolayers previously stimulated or not with TNFα (20 ng/ml; Sigma-Aldrich) for 24 h.

Leukocyte adhesion was determined after 7 min at 0.5 dyn/cm^2^. Experiments were carried out in heparinized whole blood, incubated or not with EDTA (10 mM, for 15 min, 37°C; PanReac AppliChem GmbH) to evaluate the contribution of platelets to leukocyte adhesion (Postea et al., 2012). Cells interacting with the surface of the endothelium were visualized and recorded (×20 objective, ×10 eyepiece) with an inverted microscope (Axio Observer A1; ZEISS International, Oberkochen, Germany). At least five fields were recorded for 10 s each. Finally, recorded images were saved on a computer for further analyses.

### Statistical Analysis

All results were analyzed using GraphPad Prism software version 8.0 (GraphPad Software, Inc., La Jolla, CA, United States). Values are expressed as individual data points, percentages or mean ± SEM when appropriate. For two-group comparisons, paired or unpaired two-tailed Student´s *t* test was used in data that passed both normality (Kolmogorov-Smirnov) and equal variance (Levene) tests, as appropriate; otherwise, the non-parametric Mann-Whitney U test was performed. For comparisons among multiple groups, one-way ANOVA followed by *post hoc* Bonferroni analysis was used in data that passed both normality and equal variance tests; otherwise, the non-parametric Kruskal-Wallis test followed by Dunn´s *post hoc* analysis was used. Data were considered statistically significant at *p* < 0.05. Additionally, some correlations between experimental findings were calculated using the Spearman correlation method.

## Results

In total, 20 patients (4 males and 16 females, aged 50.1 ± 3.0 years) and 10 age-matched controls (3 males and 7 females, aged 46.8 ± 4.2 years) were studied. Demographic, clinical and biochemical characteristics of subjects in fasting conditions are shown in Table 2. No statistically significant differences were found for age, gender, BMI, or waist circumference between the two groups (Table 2). By contrast, baseline levels of total cholesterol (TC), LDL, and apoB were significantly higher in patients than in controls (Table 2). After the OUFL challenge to both groups, only the circulating levels of TG were significantly increased in patients Table 3 and not the levels of the three clinical features of PH, apoB, LDL and TC, as described previously for other studies (Langsted et al., 2008).

**TABLE 2:** Clinical and fasting biochemical characteristics of the study groups^**1**^.

Parameter	Control volunteers (*n* = 10)	PH patients (*n* = 20)	*p*-value
Gender M/F (%)	3/7 (30.0/70.0)	4/16 (20.0/80.0)	0.95
Age (years)	46.8 ± 4.2	50.1 ± 3.0	0.54
BMI (kg/m^2^)	25.9 ± 1.1	25.8 ± 0.8	0.92
Waist circumference (cm)	85.6 ± 2.9	86.1 ± 1.8	0.90
SBP (mmHg)	115.4 ± 2.6	124.8 ± 3.6	0.11
DBP (mmHg)	73.5 ± 3.1	78.5 ± 2.7	0.28
Glucose (mg/dl)	83.8 ± 1.7	88.1 ± 1.8	0.16
TC (mg/dl)	215.6 ± 12.9	266.6 ± 9.1**	**0.004**
LDL (mg/dl)	137.0 ± 10.2	184.0 ± 6.3**	**0.0004**
TG (mg/dl)	93.5 ± 13.7	112.7 ± 8.5	0.24
HDL (mg/dl)	66.8 ± 3.6	63.6 ± 3.1	0.53
apoB (mg/dl)	94.7 ± 7.0	128.4 ± 5.2**	**0.001**
GOT (U/L)	23.0 ± 1.0	22.8 ± 1.1	0.91
GPT (U/L)	20.0 ± 3.4	18.6 ± 1.1	0.72
Creatinine (mg/dl)	0.7 ± 0.0	0.7 ± 0.0	0.83
IgG (mg/dl)	959.6 ± 61.5	954.6 ± 31.2	0.94
IgM (mg/dl)	103.6 ± 10.6	123.5 ± 14.6	0.40
IgE total (IU/L)	36.8 ± 12.2	49.0 ± 17.2	0.66

apoB, apolipoprotein B; BMI, body mass index; DBP, diastolic blood pressure; GOT, glutamic-oxalacetic transaminase; GPT, glutamate-pyruvate transaminase; HDL, high-density lipoprotein; Ig, immunoglobulin; LDL, low-density lipoprotein; PH, primary hypercholesterolemia; TC, total cholesterol; TG, triglycerides; SBP, systolic blood pressure.

^1^Data are presented as mean ± SEM.

^**^ *p* < 0.01 relative to values in the control group.

**TABLE 3:** Effect of an oral unsaturated fat load test on different biochemical characteristics and immunoglobulins in controls and in patients with primary hypercholesterolemia^**1**^.

Parameter	Study group	Fasting	4 h	*p*-value
Glucose (mg/dl)	Control volunteers	83.8 ± 1.7	83.7 ± 1.4	0.96
	PH patients	88.1 ± 1.8	88.3 ± 1.6	0.94
TG (mg/dl)	Control volunteers	93.5 ± 13.7	133.0 ± 21.0	0.13
	PH patients	112.7 ± 8.5	177.5 ± 17.9**	**0.0034**
IgG (mg/dl)	Control volunteers	959.6 ± 61.5	1002.1 ± 67.1	0.64
	PH patients	954.6 ± 31.2	958.6 ± 32.8	0.93
IgM (mg/dl)	Control volunteers	103.6 ± 10.6	105.9 ± 11.0	0.88
	PH patients	123.5 ± 14.6	123.3 ± 14.3	0.99
IgE total (IU/L)	Control volunteers	36.8 ± 12.2	37.3 ± 11.9	0.97
	PH patients	49.0 ± 17.2	50.0 ± 18.0	0.97

Ig, immunoglobulin; PH, primary hypercholesterolemia; TG, triglycerides.

^**^ *p* < 0.01 relative to values in patients in fasting conditions.

^1^ Data are presented as mean ± SEM.

### Platelet Activation and Related Soluble Markers are Reduced in Patients with Primary Hypercholesterolemia After an Oral Unsaturated Fat load

**FIGURE 1:**
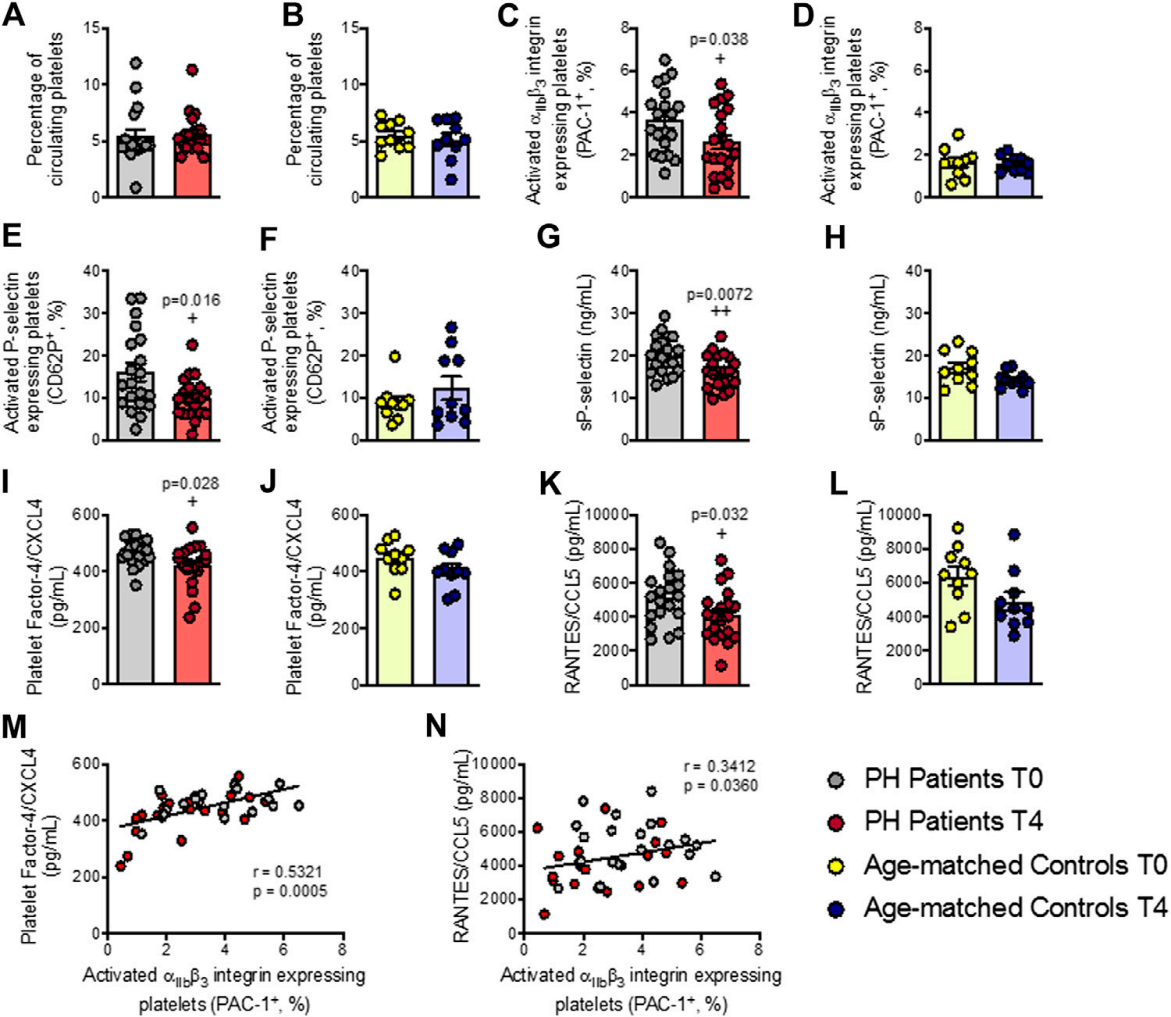
Platelet activation and related soluble markers are reduced in patients with primary hypercholesterolemia four hours after an oral unsaturated fat load but not in age-matched controls**.** Flow cytometry analysis of platelets stained with conjugated antibodies against CD41 **(A,B)**, CD41 and PAC-1 **(C,D)**, and CD41 and P-selectin **(E,F)**. Results are presented as the percentage of positive cells. sP-selectin **(G,H)**, PF-4/CXCL4 **(I**,**J)**, and RANTES/CCL5 **(K**,**L)** plasma levels (ng/mL or pg/mL) were measured by ELISA (*n* = 20 PH patients) and 10 age-matched controls. Values are expressed as mean ± SEM. +*p* < 0.05 or ++*p* < 0.01 relative to values in the PH group at time 0 (T0). Positive correlations between the percentage of platelets expressing PAC-1 and plasma levels of PF-4/CXCL4 **(M)** and RANTES/CCL5 **(N)** in PH patients. Data sets B, C, D, E, G H, J, K, and L were compared using two-tailed Student’s t-test; data sets (A,F), and I were compared using Mann-Whitney U-test; correlations M and N were calculated by the Spearman correlation method. PF-4, platelet factor-4; PH, primary hypercholesterolemia; RANTES, regulated upon activation, normal T cell expressed and secreted; sP-selectin, soluble P-selectin; T0, time 0; T4, time 4.

No changes were observed in the percentage of circulating platelets in either group after the OUFL (Figures 1A,B). By contrast, the percentage of activated platelets (PAC-1^+^ and P-selectin/CD62P^+^) was significantly lower after the OUFL in patients with PH (Figures 1C,E), but not in age-matched controls (Figures 1D,F). Since P-selectin expression on the platelet surface can be cleaved and released into circulation as soluble P-selectin (sP-selectin), we determined its circulating levels in plasma, finding that levels were significantly lower after OUFL challenge in the PH group but not in the control group (Figures 1G,H). Likewise, circulating plasma levels of PF-4/CXCL4 and RANTES/CCL5 – chemokines that are released upon platelet activation–were significantly lower after the OUFL in patients, but no differences were detected in controls (Figures 1I–L). Of note, we found positive correlations between circulating levels of PF-4/CXCL4 and RANTES/CCL5 and the percentage of platelets expressing PAC-1 in PH patients (Figures 1M,N, respectively).

### Neutrophil Activation and Circulating Levels of IL-8/CXCL8 Are Reduced in Patients With Primary Hypercholesterolemia After an Oral Unsaturated fat Load

**FIGURE 2:**
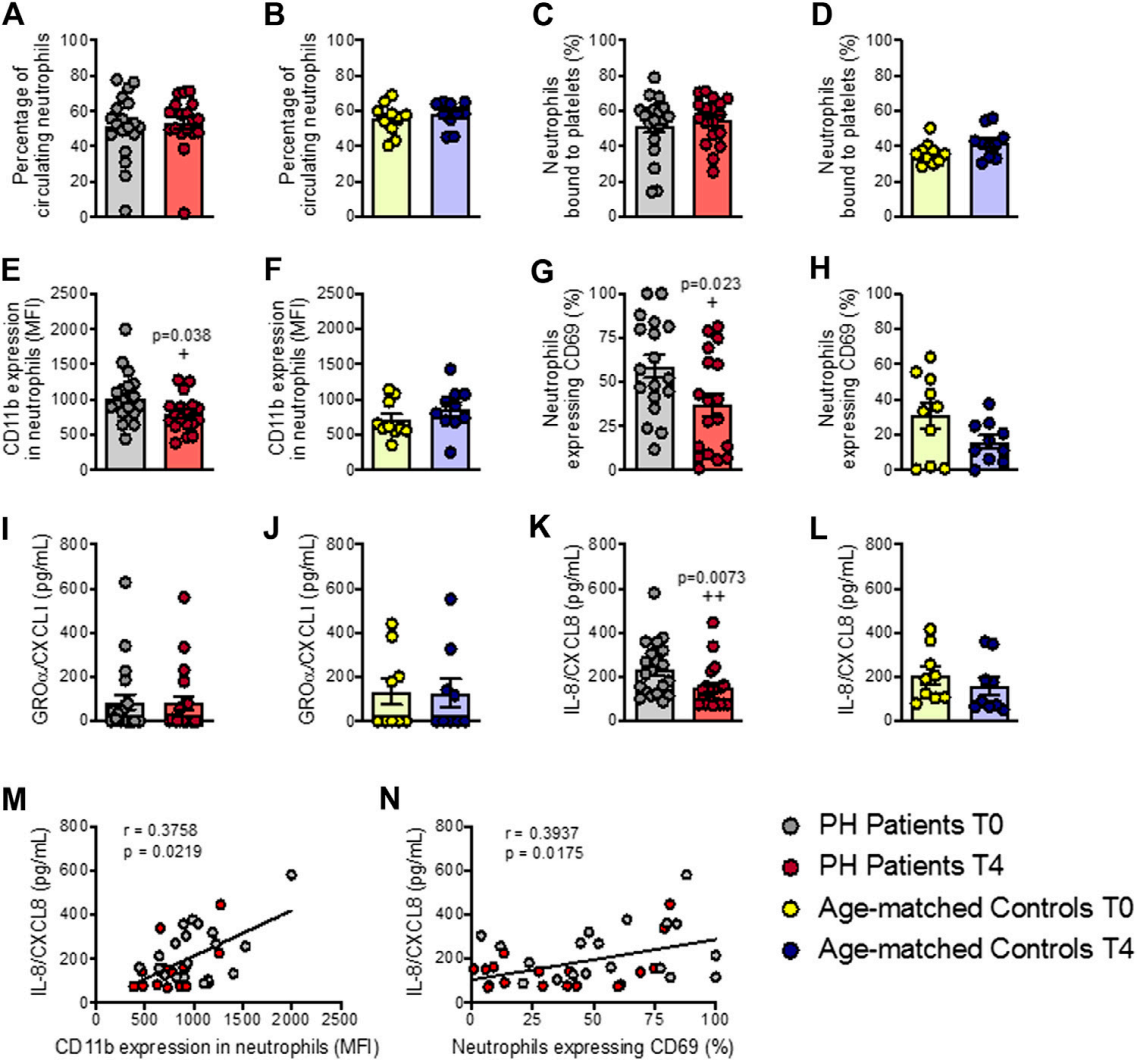
Neutrophil activation and circulating levels of IL-8/CXCL8 are reduced in patients with primary hypercholesterolemia after an oral unsaturated fat load but not in age-matched controls. Flow cytometry analysis of heparinized whole blood co-stained with specific markers for platelets and neutrophils **(A–D)**. Neutrophils were also stained for CD11b integrin **(E,F)** and CD69 **(G,H)**. Results are presented as the percentage of positive cells or median fluorescence intensity (MFI). GROα/CXCL1 **(I,J)** and IL-8/CXCL8 **(K**,**L)** plasma levels (pg/ml) were measured by ELISA (*n* = 20 PH patients) and 10 age-matched controls. Values are expressed as mean ± SEM. +*p* < 0.05 or ++*p* < 0.01 relative to values in the PH group at time 0 (T0). Positive correlations between IL-8/CXCL8 plasma levels and CD11b expression in neutrophils **(M)** and in neutrophils expressing CD69 **(N)** in PH patients. Data sets B, D, E, G, H, and L were compared using two-tailed Student’s t-test; data sets A, C, F, I, J, and K were compared using Mann-Whitney U-test; correlations M and N were calculated by the Spearman correlation method. GROα, growth-regulated oncogene α; PH, primary hypercholesterolemia; T0, time 0; T4, time 4.

We next evaluated several parameters related to the activation of different leukocyte subsets after the OUFL. No changes were observed in the percentage of circulating neutrophils or in platelet-neutrophil aggregates in either group before or after the OUFL (Figures 2A–D). By contrast, a significant reduction in the activation state of neutrophils (CD11b expression and CD69^+^) was evident after the OUFL in patients (Figures 2E,G), but not in controls (Figures 2F,H). As some chemokines can promote neutrophil activation and chemotaxis, including GROα/CXCL1 and IL-8/CXCL8, we quantified their plasma levels before and after the OUFL. No differences were found for GROα/CXCL1 in either group after the OUFL (Figures 2I,J). Plasma levels of IL-8/CXCL8 were, however, significantly lower in the PH group after the OUFL (Figures 2K,L). Also, the circulating concentration of IL-8/CXCL8 positively correlated with CD11b expression on neutrophils (Figure 2M) and with the percentage of activated neutrophils in PH patients (CD69^+^, Figure 2N).

### Circulating Mon1 Monocytes and MCP-1/CCL2 Plasma Levels Are Reduced in Patients With Primary Hypercholesterolemia After an Oral Unsaturated Fat Load

**FIGURE 3:**
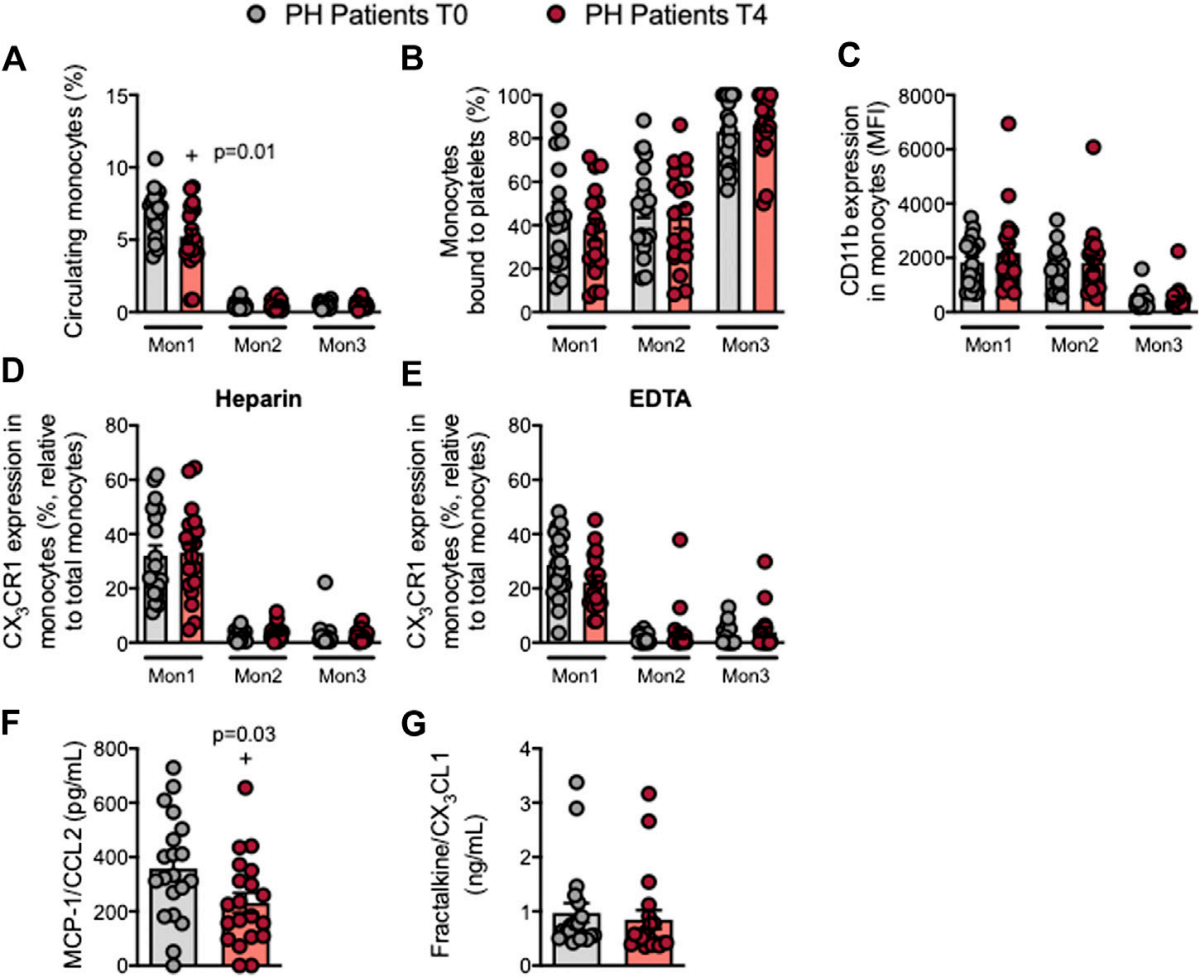
Circulating Mon1 monocytes and MCP-1/CCL2 plasma levels are reduced in patients with primary hypercholesterolemia after an oral unsaturated fat load. Flow cytometry analysis of heparinized or EDTA-treated whole blood co-stained with specific markers for platelets and Mon1, 2, and 3 monocytes **(A,B)**, CD11b integrin **(C)**, and CX_3_CR1 in heparinized **(D)** and EDTA-treated whole blood **(E)**. Results are presented as the percentage of positive cells or median fluorescence intensity (MFI). MCP-1/CCL2 **(F)** and fractalkine/CX_3_CL1 **(G)** plasma levels (ng/mL or pg/mL) were measured by ELISA (*n* = 20 PH patients). Values are expressed as mean ± SEM. +*p* < 0.05 relative to values in the PH group at time 0 (T0). Data sets A, B, C and F were compared using two-tailed Student’s t-test; data sets D, E, and G were compared using Mann-Whitney U-test. MCP-1, monocyte chemoattractant protein-1; Mon1/2/3, type 1/2/3 monocytes; PH, primary hypercholesterolemia; T0, time 0; T4, time 4.

Three monocyte subpopulations have been described in peripheral blood based on their differential expression of the cell surface markers CD14, CD16 and CCR2 (as described in Supplementary Table S2). Following the OUFL, a significant decrease in the percentage of circulating type 1 monocytes (Mon1) was observed in patients but not in controls (Figure 3A; Supplementary Figure S7A), whereas the percentage of Mon2 and 3 monocytes did not differ between groups before or after the OUFL (Figure 3A; Supplementary Figure S7A). Likewise, platelet-monocyte aggregates, CD11b integrin and CX_3_CR1 expression and plasma concentrations of fractalkine/CX_3_CL1 were unchanged by the OUFL in either group (Figures 3B–E,G; Supplementary Figures S7B–E,G). Notably, the levels of MCP-1/CCL2 were significantly reduced by the OUFL in patients but not in controls (Figure 3F; Supplementary Figure S7F).

### The Percentage of Circulating Treg Cells is Increased in Patients With Primary Hypercholesterolemia After an Oral Unsaturated Fat Load

**FIGURE 4:**
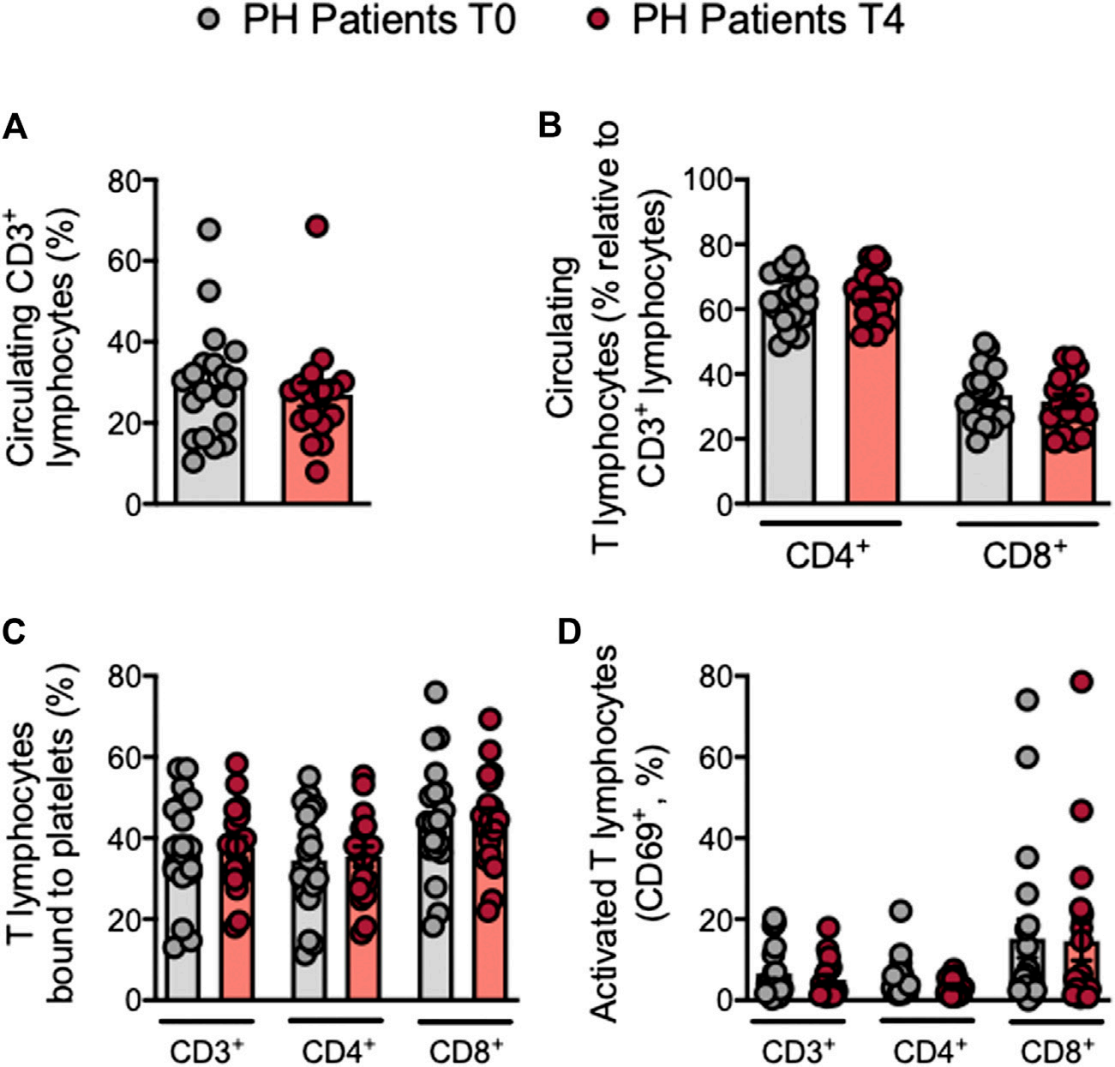
No changes in circulating T lymphocytes, platelet T lymphocyte aggregates and T lymphocyte activation in patients with primary hypercholesterolemia after an oral unsaturated fat load. Heparinized whole blood was co-stained with specific markers for platelets, CD3^+^, CD4^+^ and CD8^+^ lymphocytes **(A–C)**, and activated lymphocytes (CD69^+^) **(D)**. Results are presented as the percentage of positive cells (*n* = 20 PH patients). Values are expressed as mean ± SEM. Data sets B and C were compared using two-tailed Student’s t-test; data sets A and D were compared using Mann-Whitney U-test. PH, primary hypercholesterolemia; T0, time 0; T4, time 4.

**FIGURE 5:**
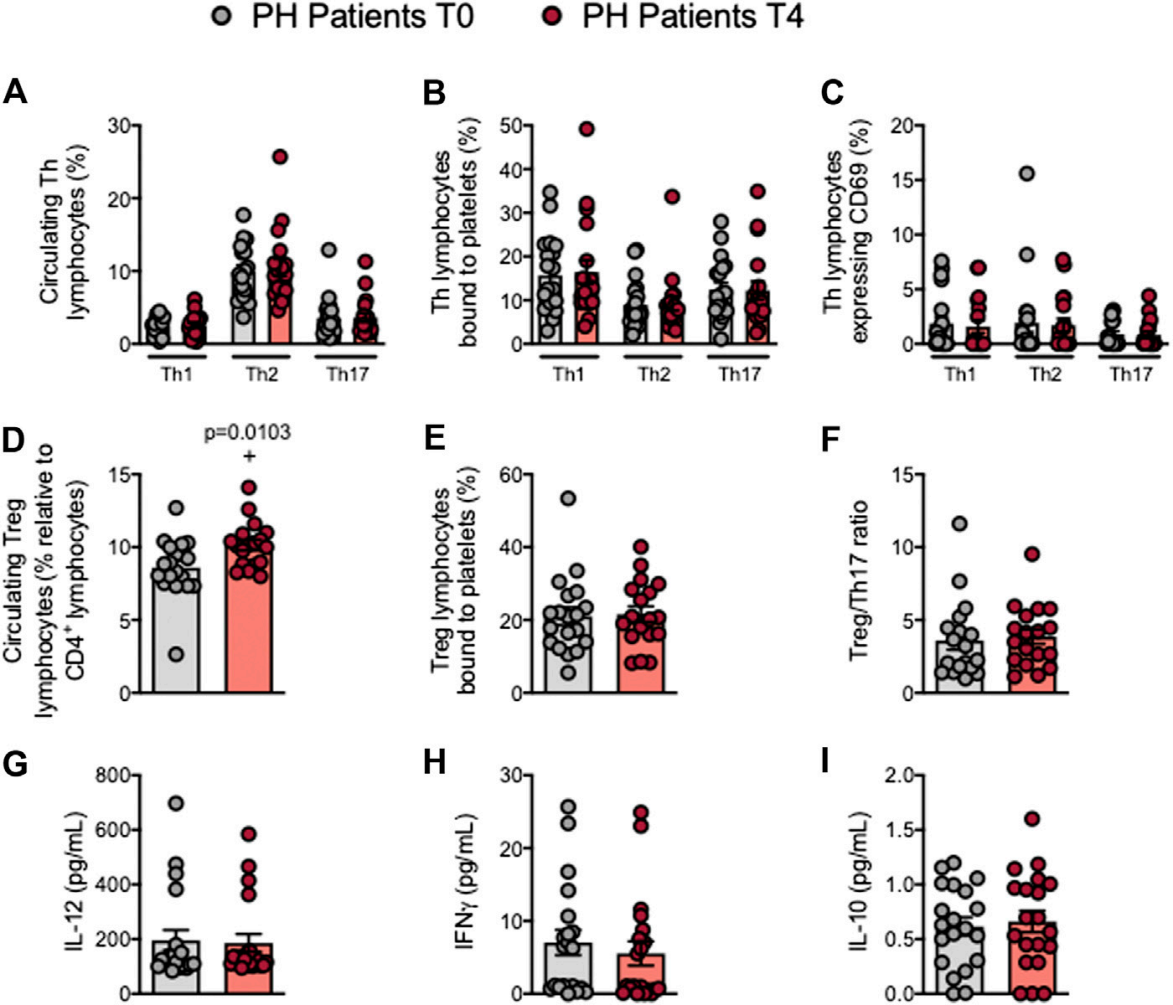
The percentage of circulating Treg cells is increased in patients with primary hypercholesterolemia after an oral unsaturated fat load. Heparinized whole blood was co-stained with specific markers for platelets and Th1, Th2, Th17, and Treg lymphocytes **(A–E)** and for activated lymphocytes (CD69^+^) **(C)**. The Treg/Th17 ratio was also determined **(F)**. Results are presented as the percentage of positive cells. IL-12 **(G)**, IFNγ **(H)**, and IL-10 **(I)** plasma levels (pg/ml) were measured by ELISA (*n* = 20 PH patients). Values are expressed as mean ± SEM. +*p* < 0.05 relative to values in the PH group at time 0 (T0). Data sets E, F, and I were compared using two-tailed Student’s t-test; data sets A, B, C, D, G, and H were compared using Mann-Whitney U-test. IFNγ, interferon γ; PH, primary hypercholesterolemia; T0, time 0; T4, time 4; Th, T helper; Treg, regulatory T cells.

Mature T cells express the general marker CD3 and either CD4 or CD8 depending on the T cell type. Following the OUFL no significant differences were found in the percentage of circulating CD3^+^, CD3^+^CD4^+^ or CD3^+^CD8^+^ cells, the percentage of platelet T lymphocyte aggregates, or the activation state of these cells in patients and controls (Figures 4A–D; Supplementry Figures S8A–D). Similarly, no differences in these parameters were detected in the different Th lymphocyte subpopulations before or after the OUFL administration in either group (Figures 5A–C; Supplementary Figures S9A–C). Interestingly, the percentage of circulating Treg lymphocytes after the OUFL was significantly higher in patients but not in controls (Figure 5D; Supplementary Figure S9D). No differences were observed in the percentage of Treg lymphocyte-platelet aggregates, the Treg/Th17 ratio or the plasma levels of different soluble markers associated with T lymphocytes (IL-12, IFNγ, or IL-10) before or after the OUFL in either group (Figures 5E–I; Supplementary Figures S9E–I).

### Circulating Levels of TNFα Are Reduced in Patients With Primary Hypercholesterolemia After an Oral Unsaturated Fat Load

**FIGURE 6:**
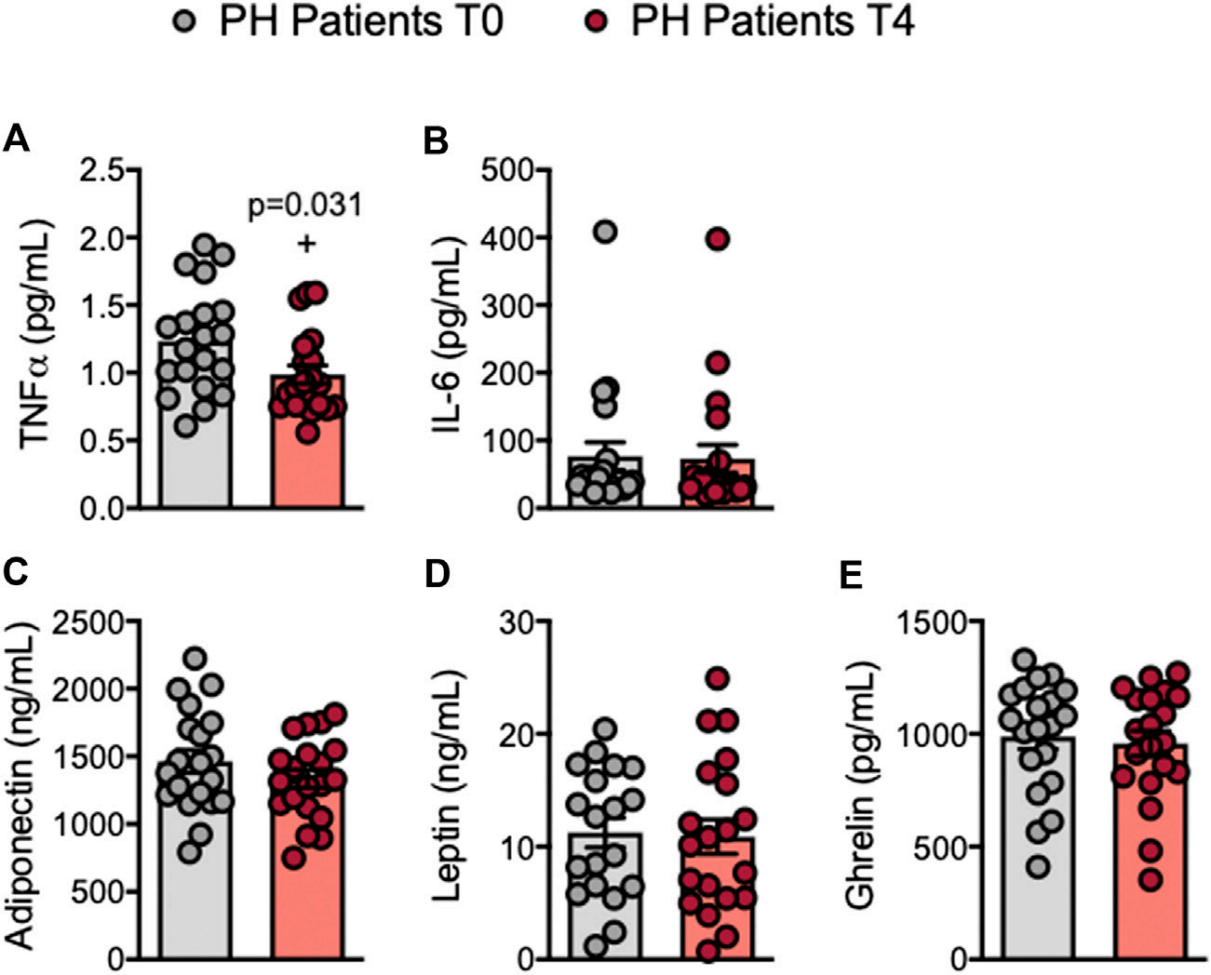
Circulating levels of TNFα are reduced in patients with primary hypercholesterolemia after an oral unsaturated fat load**.** TNFα **(A)**, IL-6 **(B)**, adiponectin **(C)**, leptin **(D)**, and ghrelin **(E)** plasma levels (ng/mL or pg/mL) were measured by ELISA (*n* = 20 PH patients). Values are expressed as mean ± SEM. +*p* < 0.05 relative to values in the PH group at time 0 (T0). Data sets A, C, D, and E were compared using two-tailed Student’s t-test; data set B was compared using Mann-Whitney U-test. PH, primary hypercholesterolemia; T0, time 0; T4, time 4.

Increased plasma levels of TNFα and IL-6 have been reported in patients with PH (Sampietro et al., 1997; Real et al., 2010; Holven et al., 2014; Collado et al., 2018a). We found that circulating plasma TNFα levels were significantly lower after the OUFL in patients and similar to those found in controls, which did not change 4 h after the administration (Figure 6A; Supplementary Figure S10A). Conversely, plasma concentrations of IL-6, adiponectin, leptin or ghrelin were unchanged after the OUFL in both groups (Figures 6B–E; Supplementary Figures S10B–E).

### Circulating Platelet-Leukocyte Aggregates and Leukocytes From Patients With Primary Hypercholesterolemia Show Reduced Adhesiveness to TNFα-Stimulated Endothelial Cells After an Oral Unsaturated Fat Load

**FIGURE 7:**
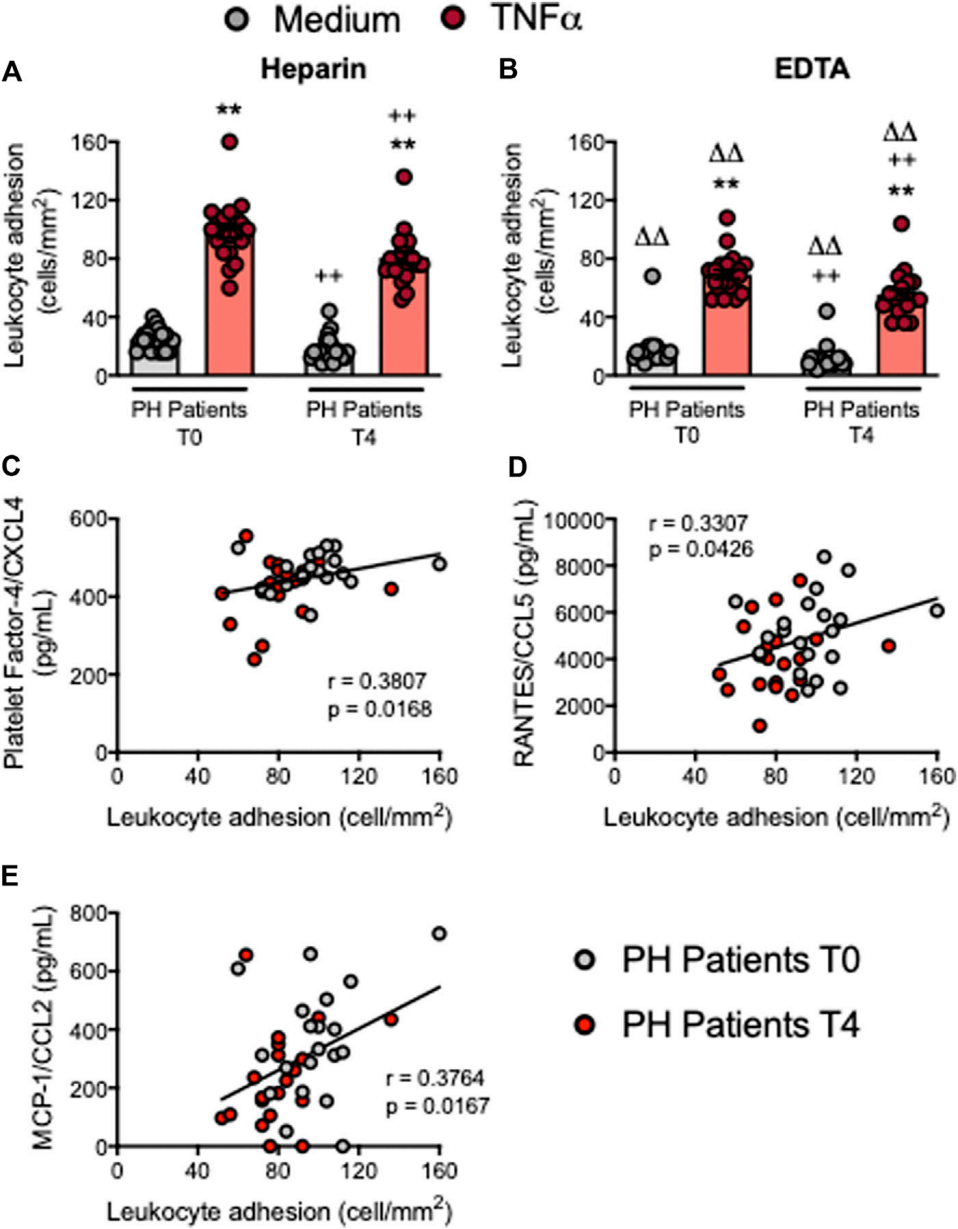
Circulating platelet-leukocyte aggregates and leukocytes from patients with primary hypercholesterolemia display lower adhesiveness to TNFα-stimulated HUAEC after an oral unsaturated fat load. HUAEC were stimulated or not with TNFα (20 ng/ml) for 24 h. Subsequently, whole blood from all patients, incubated without **(A)** or with EDTA **(B)**, was perfused across the endothelial monolayers for 7 min at 0.5 dyn/cm^2^ and leukocyte adhesion quantified (cells/mm^2^). Values are expressed as mean ± SEM (*n* = 20 PH patients). **p < 0.01 relative to values in the medium-only group; ++*p* < 0.01 relative to the respective values at time 0 (T0); ∆∆*p* < 0.01 relative to the respective values in the heparin group. Positive correlations between leukocyte adhesion and plasma levels of PF-4/CXCL4 **(C)**, RANTES/CCL5 **(D)**, and MCP-1/CCL2 **(E)**. Data sets A and B were compared using Kruskal-Wallis (Dunn’s *post hoc*) test; correlations C, D and E were calculated by the Spearman correlation method. HUAEC, human umbilical artery endothelial cells; MCP-1, monocyte chemoattractant protein-1; PF-4, platelet factor-4; PH, primary hypercholesterolemia; RANTES, regulated upon activation, normal T cell expressed and secreted; T0, time 0; T4, time 4.

Endothelial dysfunction is the earliest stage of atherogenesis and is characterized by an increase in the adhesiveness of leukocytes to the endothelium, and their subsequent migration to the arterial subendothelial space (Landmesser et al., 2004). We previously demonstrated that the plasma levels of TNFα are elevated in patients with PH and that the adhesion of platelet-leukocyte aggregates and leukocytes from these patients to dysfunctional arterial endothelium (TNFα-stimulated) is enhanced when compared with age-matched controls (Collado et al., 2018a). We performed the flow chamber adhesion analysis before and following the OUFL, finding that the number of platelet-leukocyte aggregates (heparin) or platelet-free leukocytes (EDTA) adhered to the unstimulated or TNFα-stimulated arterial endothelium was reduced after the OUFL in patients (Figures 7A,B) but not in controls (Supplementary Figures S11A,B). Of note, we found positive correlations between patient leukocyte adhesion and PF-4/CXCL4 (Figure 7C), RANTES/CCL5 (Figure 7D), and MCP-1/CCL2 (Figure 7E) plasma levels.

## Discussion

PH is characterized by elevated plasma levels of cholesterol–specifically, LDL and apoB–which contribute to the development of atherosclerosis and associated ischemic events (Sampietro et al., 1997; Chironi et al., 2006; Real et al., 2010; Holven et al., 2014; Cortes et al., 2016; Collado et al., 2018a; Hansen et al., 2019). In addition, there is increasing evidence that systemic inflammation is the main driver of premature atherosclerosis (Catapano et al., 2017) and is a component of PH (Langslet et al., 2015; Barale et al., 2018; Collado et al., 2018a). In the present study, we have extensively analyzed the acute impact (4 h) of an OUFL containing 58% oleic acid and 20% linoleic acid (Supplementary Table S1) on the systemic inflammatory response associated with PH. We show that an OUFL challenge beneficially modulates different immune players, reduces the levels of inflammatory cytokines and chemokines and impairs a prominent feature of the atherogenesis–the adhesiveness of leukocytes to the dysfunctional arterial endothelium. In agreement with our findings, oleic acid has been shown to protect against CVD and insulin resistance, and to improve endothelial dysfunction in response to pro-inflammatory signals (Perdomo et al., 2015). And in the same line, dietary intake of linoleic acid is inversely associated with the risk of coronary heart disease (Farvid et al., 2014).

Inflammation triggers platelet activation, which in turn plays an important role in several processes such as homeostasis (Manne, 2017; Periayah et al., 2017) and thrombosis (Mancuso and Santagostino, 2017). Activated platelets are now also recognized as essential immune-modulators (Lam et al., 2015) by their expression of specific cell adhesion molecules such as P-selectin, which plays a crucial role in the recruitment of leukocytes to the inflammatory site. Additionally, they can release various inflammatory chemokines, including PF-4/CXCL4 or RANTES/CCL5 which can be deposited in the endothelium to stimulate monocyte and lymphocyte recruitment (von Hundelshausen and Schmitt, 2014). Patients with PH show a pro-thrombotic state characterized by increased platelet activation, which is reflected by the presence of P-selectin^+^ and PAC-1^+^ platelets (Collado et al., 2018a). We found that a lipid OUFL challenge significantly reduced platelet activation in patients, pointing to the potential anti-thrombotic effects of the intervention. Additionally, OUFL reduced the circulating levels of several inflammatory mediators linked to platelet activation including sP-selectin, PF-4/CXCL4 and RANTES/CCL5, again suggesting that in postprandial state this treatment may impair the pro-thrombotic state associated with PH and the progression of atherogenesis (von Hundelshausen and Schmitt, 2014).

To understand the immune state of the PH environment, we surveyed different leukocyte subtypes following OUFL challenge. Neutrophils are known to be one of the major players in acute inflammation (Kolaczkowska and Kubes, 2013), as they express integrin CD11b/CD18 that is up-regulated upon activation and promotes leukocyte adhesion and transmigration across the vascular endothelium through its interaction with its cognate ligands intercellular adhesion molecule (ICAM)-1 and ICAM-2 (Diacovo et al., 1996). Our analysis showed that while there no differences were evident in the percentage of circulating neutrophils and neutrophil-platelet aggregates after the OUFL in patients, there was a significant reduction in their activation state (CD11b expression and CD69^+^). This was accompanied by a clear reduction in the circulating levels of IL-8/CXCL8, which induces neutrophil activation and chemotaxis (Kolaczkowska and Kubes, 2013). Both outcomes were positively correlated, thus indicating an improvement in the immune state of patients with PH and ameliorating the proatherogenic status.

Human circulating monocytes comprise a heterogeneous cell population that is commonly classified into three subtypes: classical CD14^++^CD16^*−*^CCR2^+^ (Mon1), intermediate CD14^++^CD16^+^CCR2^+^ (Mon2), and non-classical CD14^+^CD16^++^CCR2^*−*^ (Mon3) (Weber et al., 2016), with the Mon1 subtype more commonly known as classical or inflammatory monocytes. We found that an OUFL led to a significant reduction in the percentage of circulating Mon1 monocytes. Interestingly, there is evidence to support that adults with FH have a pro-inflammatory imbalance in circulating monocyte subpopulations (Mon1) (Fadini et al., 2014). Although different studies in humans have noted increases in circulating CD16^+^ monocytes in CVD (Kratofil et al., 2017), we observed no changes in Mon2 and Mon3 populations, monocyte activation state (CD11b expression) or CX_3_CR1 expression after the OUFL. By contrast, MCP-1/CCL2 circulating levels were significantly reduced in patients with PH after the OUFL, confirming a previous report in patients with FH (Cortes et al., 2016). MCP-1/CCL2 mainly recruits Mon1 and Mon2 monocytes to inflammatory sites through interaction with its CCR2 receptor (Weber et al., 1999; Deshmane et al., 2009), and this inflammatory axis has been widely associated with CVD development (Franca et al., 2017).

T lymphocyte analysis revealed no changes in total T or Th lymphocytes after the OUFL in patients with PH but a significant increase in the percentage of Treg lymphocytes, which might contribute to the anti-inflammatory environment created by this intervention. However, neither the Treg/Th17 ratio nor the circulating levels of IL-10 were improved by the OUFL, although it is tempting to speculate that changes in these parameters might be evident at later time points. Of note, plasma levels of TNFα were significantly reduced in patients after the OUFL being normalized to control subjects’ levels. In this regard, a prior study in FH found that a similar intervention decreased the circulating levels of several inflammatory chemokines including macrophage inflammatory protein (MIP)-1α, MIP-1β, and interferon γ-induced protein-10 (IP-10)/CXCL10, among others, with values close to those found in control subjects (Cortes et al., 2016).

Finally, we used the dynamic flow chamber to explore the functional consequences of platelet-leukocyte-endothelium (heparin) or leukocyte-endothelium (EDTA) interactions. We previously showed that adhesion of platelet-leukocyte aggregates to HUAEC stimulated or not with TNFα is significantly higher in patients with PH than in controls (Collado et al., 2018a). When these parameters were evaluated after the OUFL challenge, we found lowered adhesion of both platelet-leukocyte aggregates (heparin) and platelet-free leukocytes (EDTA) to dysfunctional arterial endothelium. The reduction in leukocyte adhesion is likely the consequence of several of the aforementioned experimental observations. First, the reduced activation state of neutrophils (CD11b/CD18 integrin down-regulation) can lead to decreased interactions with the constitutively or inducible (TNFα-stimulated) expressed endothelial ICAM-1. Second, since activated platelets can mediate the endothelial adhesion of circulating leukocytes–a characteristic feature of the dysfunctional endothelium (Rius et al., 2013; Landmesser et al., 2004; Marques et al., 2017; Collado et al., 2018a; Collado et al., 2018b; Furio et al., 2018) – their decreased activation may alter leukocyte arrest. Third, a reduction in the percentage of circulating inflammatory (classical) monocytes (Mon1) results in diminished monocyte adhesion. Finally, the decreased levels of circulating chemokines may also affect the adhesion of leukocytes to endothelium in patients with PH. Supporting this concept, neutralization of CCL2 activity was found to decrease the endothelial arrest of Mon1 monocytes (Marques et al., 2019). Likewise, platelet deposition of RANTES/CCL5 in the endothelium can trigger monocyte arrest (von Hundelshausen et al., 2001) and PF-4/CXCL4 has multiple atherogenic activities and synergizes with CCL5 (von Hundelshausen and Schmitt, 2014). Leukocyte adhesion in this setting positively correlates with the plasma levels of these chemokines.

Our study has limitations. First, to date the acute intervention of the OUFL do not allow us to extrapolate these results to those with a long-term intervention and second, Supracal® cannot be considered a physiological ingestion of fat. Nevertheless, preclinical studies in animal models of hypercholesterolemia will be designed to evaluate the long-term effects of the OUFL.

In summary, administration of an OUFL has beneficial acute effects on the postprandial pro-thrombotic and pro-inflammatory state of PH patients. Further long-term studies are, however, warranted. Our findings indicate that the modulation of the cellular and soluble inflammatory components in PH might be crucial to prevent further cardiovascular complications.

## Data Availability

The original contributions presented in the study are included in the article/Supplementary Material, further inquiries can be directed to the corresponding authors.
