# Supplementary material for: Oral Unsaturated Fat Load Impairs Postprandial Systemic Inflammation in Primary Hypercholesterolemia Patients
Source: Front Pharmacol. 2021 Apr 20;12:656244. doi: 10.3389/fphar.2021.656244 (PMC8093814; doi:10.3389/fphar.2021.656244)
Supplement: Supplementary file 1 [file datasheet1.docx]

Supplementary Material

**Supplementary Table S1**. **Fatty acid composition of Supracal®** (Pedro, Martinez-Hervas et al. 2013)

| **g/100 g fatty acids** | |
| --- | --- |
| **Lauric** (C_12_) | <1 |
| **Myristic** (C_14_) | <1 |
| **Palmitic** (C_16_) | 10 |
| **Stearic** (C_18_) | 3 |
| **Oleic** (C_18:1_) | 58 |
| **Linoleic** (C_18:2_) | 20 |
| **α-linoleic** (C_18:3_) | <1 |
| **Arachidonic** (C_20_) | 1 |
| **Eicosanoic** (C_20:1_) | 1 |
| **Behenic** (C_22_) | 3 |
| **Lignoceric** (C_24_) | 1 |

Pedro, T., S. Martinez-Hervas, C. Tormo, A. B. Garcia-Garcia, G. Saez-Tormo, J. F. Ascaso, F. J. Chaves, R. Carmena and J. T. Real (2013). "Oxidative stress and antioxidant enzyme values in lymphomonocytes after an oral unsaturated fat load test in familial hypercholesterolemic subjects." Transl Res **161**(1): 50-56.

**Supplementary
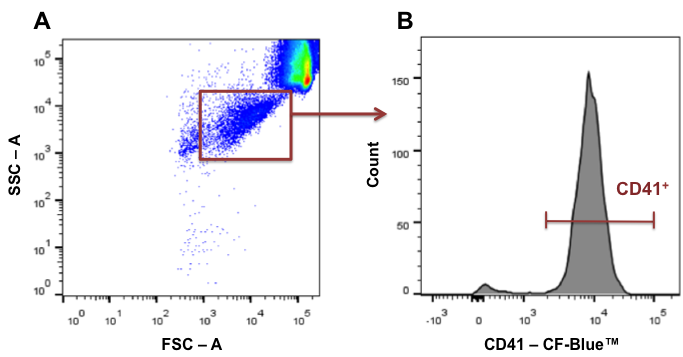
Figure S1. Gating strategy for human platelets in citrated whole blood according to morphological properties and CD41 detection by flow cytometry.** Platelets were gated according to a low side scatter (SSC*−*­A) and forward scatter (FSC*−*­A), shown in a logarithmic scale (**A**) and defined as CD41^+^ population (**B**).

**Supplementary Figure S2. Gating strategy for human neutrophils in whole blood according to morphological properties and CD16 expression by flow cytometry.** Neutrophils were selected by CD45 labeling and morphology (high SSC*−***­**A; **A**). A CD16 antibody was then used to detect neutrophils (CD16^+^; **B**). In heparinized whole blood, neutrophil-platelet complexes were selected as a CD16^+^CD41^+^ population, and platelet-free neutrophils were gated as CD16^+^CD41*^−^* from blood incubated with EDTA (**C**).

Supplementary Table S2. Differential markers of monocyte subpopulations

| **Markers** | **Cellular subpopulation** |
| --- | --- |
| CD14^++^CD16*^−^*CCR2^+^ | Type 1 monocytes (Mon1) |
| CD14^++^CD16^+^CCR2^+^ | Type 2 monocytes (Mon2) |
| CD14^+^CD16^++^CCR2*^−^* | Type 3 monocytes (Mon3) |

**Supplementary** **Figure S3. Gating strategy for human monocyte subpopulations detection in whole blood by flow cytometry.** First, monocytes were selected by CD14 labeling and morphology (medium SSC*−***­**A; **A**), and then by the marker CD16 (**B**). For the detection of different monocyte subpopulations, CD16 and CCR2 markers were used (**B**–**F**). Type 1 monocytes (Mon1) were selected as CD14^++^CD16*^−^*CCR2^+^, type 2 monocytes (Mon2) were selected as CD14^++^CD16^+^CCR2^+^, and type 3 monocytes (Mon3) were selected as CD14^+^CD16^++^CCR2*^−^* (**C**). Platelet-Mon1 complexes were selected as CD14^++^CD16*^−^*CCR2^+^CD41^+^ population from heparinized whole blood, and platelet-free Mon1 were gated as CD14^++^CD16*^−^*CCR2^+^CD41^−^ from blood incubated with EDTA (**D**). Platelet-Mon2 complexes were selected as CD14^++^CD16^+^CCR2^+^CD41^+^ population, while platelet-free Mon2 were gated as CD14^++^CD16^+^CCR2^+^CD41^−^ (**E**). Finally, platelet-Mon3 complexes were selected as CD14^+^CD16^++^CCR2*^−^*CD41^+^ population, and platelet-free Mon3 were gated as CD14^+^CD16^++^CCR2*^−^*CD41^−^ (**F**).

**Supplementary Figure S4. Gating strategy for human T lymphocyte detection in whole blood by flow cytometry.** T lymphocytes were selected as the CD3^+^ population and with a low SSC*−***­**A (**A**). T helper (Th) lymphocytes were the CD3^+^CD4^+^ population and cytotoxic T lymphocytes were selected as CD3^+^CD8^+^ (**B**). In heparinized whole blood, T lymphocyte-platelet complexes were gated as CD3^+^CD41^+^ populations, while platelet-free T lymphocytes were selected as CD3^+^CD41^−^ from blood incubated with EDTA (**C**). Th lymphocyte-platelet complexes were selected as CD3^+^CD4^+^CD41^+^ populations, while platelet-free Th lymphocytes were gated as CD3^+^CD4^+^CD41^−^ (**D**). Finally, cytotoxic T lymphocyte-platelet complexes were selected as CD3^+^CD8^+^CD41^+^ populations, while platelet-free cytotoxic T lymphocytes were gated as CD3^+^CD8^+^CD41^−^ (**E**).

Supplementary Table S3. Differential markers of Th^1^ lymphocyte subpopulations

| **Markers** | **Cellular subpopulation** |
| --- | --- |
| CD4^+^CXCR3^+^CCR6*^−^* | Type 1 T helper (Th1) |
| CD4^+^CXCR3*^−^*CCR6*^−^* | Type 2 T helper (Th2) |
| CD4^+^CXCR3*^−^*CCR6^+^ | Type 17 T helper (Th17) |

**^1^**Th: T helper lymphocyte.

**Supplementary Figure S5. Gating strategy for human T helper lymphocytes detection in whole blood by flow cytometry.** T helper (Th) lymphocytes were selected as the CD4^+^ population and with a low SSC*−***­**A (**A**). Th lymphocyte subpopulations were detected with the markers CCR6 and CXCR3 (**B**). In heparinized whole blood, Th1 lymphocyte-platelet complexes were selected as CD4^+^CXCR3^+^CCR6^−^CD41^+^, while platelet-free Th1 lymphocytes were gated as CD4^+^CXCR3^+^CCR6^−^CD41^−^ in blood treated with EDTA (**B** and **C**). Th2 lymphocyte-platelet complexes were selected as CD4^+^CXCR3^−^CCR6^−^CD41^+^ and platelet-free Th2 lymphocytes were gated as CD4^+^CXCR3^−^CCR6^−^CD41^−^ (**B** and **D**). Finally, Th17 lymphocyte-platelet complexes were selected as CD4^+^CXCR3^−^CCR6^+^CD41^+^, while Th17 lymphocyte-platelet-free complexes were selected as CD4^+^CXCR3^−^CCR6^+^CD41^−^ in blood treated with EDTA (**B** and **E**).

**Supplementary Figure S6. Gating strategy for human regulatory T lymphocytes detection in whole blood by flow cytometry.** T regulatory (Treg) lymphocytes were selected as the CD4^+^ population and with a low SSC*−***­**A (**A**). Treg lymphocytes were detected with the markers CD127 and CD25 (**B**). Treg lymphocyte-platelet complexes were selected as the CD4^+^CD127^−^CD25^+^CD41^+^ population from heparinized whole blood, and platelet-free lymphocytes were gated as CD4^+^CD127^−^CD25^+^CD41^−^ from blood incubated with EDTA (**C**).

**Supplementary Figure S7.** **No significant changes were observed in the different monocyte subpopulations or in plasma levels of MCP-1/CCL2 and fractalkine/CX_3_CL1 in control subjects 4 hours after OUFL administration.** Flow cytometry analysis of heparinized whole blood co-stained with specific markers for platelets and Mon1, 2 and 3 monocytes (**A** and **B**), CD11b integrin (**C**), and CX_3_CR1 in heparinized (**D**) and EDTA-treated whole blood (**E**). Results are presented as percentage of positive cells or median fluorescence intensity (MFI). MCP-1/CCL2 (**F**) and fractalkine/CX_3_CL1 (**G**) plasma levels (pg/mL or ng/mL) were measured by ELISA (*n* = 10 control subjects). Values are expressed as mean ± SEM. Data sets A, B, C and D were compared using two-tailed Student’s t-test; data sets E, F and G were compared using Mann-Whitney U-test. MCP-1: monocyte chemoattractant protein-1; Mon1/2/3: type 1/2/3 monocytes; OUFL: oral unsaturated fat load; T0: time 0; T4: time 4.

**Supplementary Figure S8.** **No significant changes in circulating T lymphocytes, platelet-T lymphocyte aggregates and T lymphocyte activation were observed in control subjects 4 hours after OUFL administration.** Heparinized whole blood was co-stained with specific markers for platelets, CD3^+^, CD4^+^, and CD8^+^ lymphocytes (**A**, **B** and **C**) as well as CD69 (**D**). Results are presented as the percentage of positive cells (*n* = 10 control subjects). Values are expressed as mean ± SEM. Data sets A, B and C were compared using two-tailed Student’s t-test; data set D was compared using Mann-Whitney U-test. OUFL: oral unsaturated fat load; T0: time 0; T4: time 4.

**Supplementary Figure S9. No significant changes in circulating T lymphocytes, platelet-T lymphocyte aggregates or T lymphocyte activation were detected in control subjects 4 hours after OUFL administration.** Heparinized whole blood was co-stained with specific markers for platelets and Th1, Th2, Th17 and Treg lymphocytes (**A**, **B**, **D** and **E**) as well as for CD69 (**C**). The Treg/Th17 ratio was also determined (**F**). Results are presented as percentage of positive cells. IL-12 (**G**), IFNγ (**H**), and IL-10 (**I**) plasma levels (pg/mL) were measured by ELISA (*n* = 10 control subjects). Values are expressed as mean ± SEM. Data sets A, D, E, F and I were compared using two-tailed Student’s t-test; data sets B, C, G and H were compared using Mann-Whitney U-test. IFNγ: interferon γ; OUFL: oral unsaturated fat load; T0: time 0; T4: time 4; Th: T helper; Treg: regulatory T cells.

**Supplementary Figure S10. No significant changes in cytokine or adipokine profile were detected in control subjects 4 hours after OUFL administration.** TNFα (**A**), IL-6 (**B**), adiponectin (**C**), leptin (**D**), and ghrelin (**E**) plasma levels (pg/mL or ng/mL) were measured by ELISA (*n* = 10 control subjects). Values are expressed as mean ± SEM. Data sets A, C and E were compared using two-tailed Student’s t-test; data sets B and D were compared using Mann-Whitney U-test. OUFL: oral unsaturated fat load; T0: time 0; T4: time 4.

**Supplementary Figure S11.** **No significant changes were detected in circulating platelet-leukocyte aggregates or leukocyte adhesion to TNFα-stimulated HUAEC in control subjects 4 hours after OUFL administration.** HUAEC were stimulated or not with TNFα (20 ng/mL) for 24 h. Subsequently, whole blood from patients and controls, incubated without (**A**) or with EDTA (**B**), was perfused across endothelial monolayers for 7 min at 0.5 dyn/cm^2^ and leukocyte adhesion quantified (cells/mm^2^). Values are expressed as mean ± SEM (*n* = 10 control subjects). ***p* <0.01 relative to values in the medium group; ∆*p* <0.05 or ∆∆*p* <0.01 relative to respective values in the heparin group. Data sets A and B were compared using one-way ANOVA (Bonferroni *post hoc*) test. HUAEC: human umbilical artery endothelial cells; MCP-1: monocyte chemoattractant protein-1; OUFL: oral unsaturated fat load; PF-4: platelet factor-4; RANTES: regulated upon activation, normal T cell expressed and secreted; T0: time 0; T4: time 4.
